# Anodal tDCS and virtual reality gait rehabilitation in individuals with chronic stroke: a case series report

**DOI:** 10.3389/fstro.2025.1489031

**Published:** 2025-01-30

**Authors:** Aracely Marks, Shelley Oliveira Barbosa, Daniella Napoli, Susan E. D'Andrea

**Affiliations:** Motion VR Biomechanics Laboratory, Department of Kinesiology, University of Rhode Island, Kingston, RI, United States

**Keywords:** stroke, virtual reality, tDCS, gait, rehabilitation

## Abstract

**Background:**

Stroke is a principal cause of long-term disability worldwide, significantly impairing motor function, including gait and mobility. Conventional physical therapy, primarily focusing on repetitive, task-specific exercises, often falls short in addressing the complex rehabilitative needs of stroke survivors. Emerging technologies such as virtual reality (VR) and transcranial direct current stimulation (tDCS) have shown potential to enhance neuroplasticity and functional recovery, suggesting that their combined use could offer a novel pathway for stroke rehabilitation.

**Objective:**

This study evaluated the efficacy of an integrated VR and tDCS treadmill training protocol in improving gait and mobility outcomes among individuals with chronic stroke.

**Methods:**

Five chronic stroke patients were recruited for this study. Participants were randomly assigned to receive either anodal tDCS or sham stimulation in conjunction with VR treadmill training. The anodal stimulation was targeted at the ipsilesional motor cortex, specifically over the primary motor cortex (M1) area corresponding to the C3/C4 locations in the 10–20 EEG system. The intervention consisted of 10 30-min sessions over 2 weeks. Clinical assessments, including the Dynamic Gait Index (DGI), Berg Balance Scale (BBS), 10-meter Walk Test (10MWT), and the Timed Up and Go Test (TUG) were conducted pre-intervention, immediately post-intervention, and at a 2-week follow-up.

**Results:**

All participants demonstrated improvements in the clinical measures post-intervention, irrespective of whether they received anodal tDCS or sham stimulation. Notably, clinically significant improvements, defined by an improvement greater or equal to the established minimal clinically important differences (MCIDs), were observed in DGI scores for four participants, suggesting enhanced gait functionality.

**Conclusion:**

The combined VR and tDCS interventions promise to improve gait and mobility in chronic stroke survivors. While the observed improvements were not distinctly attributed to tDCS, the role of VR training was notably beneficial. These preliminary findings underscore the potential of integrating emerging technologies in stroke rehabilitation and highlight the need for future research with larger cohorts to explore the distinct contributions of each modality and validate this integrative approach.

## Introduction

Stroke is a leading cause of long-term disability, often resulting in motor deficits like weakness, spasticity, poor coordination, and balance issues, which impair gait (Langhorne et al., 2011). Post-stroke rehabilitation is essential for mobility and independence but relies heavily on structured, repetitive exercises (Pollock et al., 2014) which may lack the engagement needed for sustained practice and motor learning (Winstein et al., 2016). Challenges such as therapist availability and the cost of long-term care highlight the need for cost-effective, innovative solutions to support home-based exercise (Cramer et al., 2011).

Virtual reality (VR) rehabilitation addresses these limitations, offering engaging, variable, and intensive rehabilitation options. VR therapy integrates visual and sensory feedback to create an immersive and motivating environment that can drive neuroplastic changes through an enriched experience (Laver et al., 2011). Virtual environments simulate real-world tasks and challenges that can be adapted to individual patient needs and progress (Crosbie et al., 2007). Anodal transcranial direct current stimulation (tDCS), a form of non-invasive brain stimulation, has been shown to enhance cortical excitability, potentially facilitating the relearning of motor skills and expediting recovery, which may be pivotal in restoring motor function post-stroke (Stagg and Johansen-Berg, 2013). Using the engaging and adaptable nature of VR and the neuroplasticity promoting effects of tDCS, this integrative approaches could represent a new path for post-stroke motor recovery (Fregni and Pascual-Leone, 2007).

A considerable amount of research has explored the efficacy of VR rehabilitation in stroke. Recent literature shows the VR training can improve balance and fall risk (Kannan et al., 2019; Lee et al., 2024; Zhang et al., 2021) and mobility and gait (Anwar et al., 2021; De Keersmaecker et al., 2023; Gibbons et al., 2016; Lin et al., 2023). Additionally, by delivering real-life environments, VR training can accelerate the transfer of skills to activities of daily living (Aderinto et al., 2023).

This paper presents a series of five patients who receive VR training to improve gait and mobility after a stroke.

## Methods

### Study design

Participants were randomized into two groups: VR therapy with anodal tDCS or VR therapy with sham tDCS. Baseline assessments related to clinical gait and mobility were completed before the VR training began. This was followed by 10 VR training sessions over 2 weeks. Clinical assessments were repeated within 48 h of the final training session and 2 weeks post-training.

### Participants

Five participants were enrolled in this study and provided written informed consent prior to participating in the study, which was conducted in accordance with ethical standards of the Institutional Review Board of the University of Rhode Island [# 1587302]. Participants were stroke patients between 18 and 75 years old, at least 6 months post-stroke, could walk continuously for 10 min, maintain a standing posture for at least 5 min and scored ≤ 23 on the lower extremity Fugl-Meyer Motor Assessment (Kwong and Ng, 2019) indicating a presence of weakness or partial paralysis (Table 1). Participants were excluded if there was history of dementia, multiple strokes, uncontrolled diabetes, presence of severe cognitive or communicative disorders, legal blindness, presence of heart failure or COPD, orthopedic conditions involving the lower limbs that limit range of motion, implanted electronic devices, and current pregnancy during the time participating in the study.

**Table 1:** Participant demographics.

**ID**	**Sex**	**Age**	**Years post stroke**	Dominant leg^*^	**Paretic leg**	**Stroke type**	**Stroke location**	**Fugl-Meyer Score**	**Active/sham tDCS**
1	F	57	8	R	R	Hemorrhagic	LCC	22	Sham
2	M	61	9	L	R	Ischemic	LCC	17	Sham
3	M	44	20	L	R	Hemorrhagic	BG	4	Active
4	F	69	12	R	R	Ischemic	LFL	23	Active
5	M	29	7	R	R	Hemorrhagic	BG	18	Sham

LCC, left cerebral cortex; BG, basal ganglia; LFL, left frontal lobe.

^*^Self reported.

### Intervention

The tDCS system (Neuroelectronics, Barcelona, Spain) was used with a headcap, ensuring standardized electrode placement according to the 10–20 system for EEG, and sponge electrodes (25 cm^2^). Anodal stimulation was provided to the ipsilesional motor cortex, with the anode positioned over the primary cortex (M1), corresponding to the C3/C4 location on the hemisphere affected by the stroke. The cathode was placed in the supraorbital region on the contralateral side (Fp1/Fp2). This facilitated the current flow from the anode, across the motor cortex and exiting through the cathode (DaSilva et al., 2011) to enhance cortical excitability. A 2mA current was applied for 30 min during the VR treadmill training sessions. Participants in the sham group received stimulation for 30 s at the start of each session to mimic the sensation of tDCS, after which the stimulation was turned off. Participants were blinded to their group allocation.

### Virtual environment

**Figure 1:**
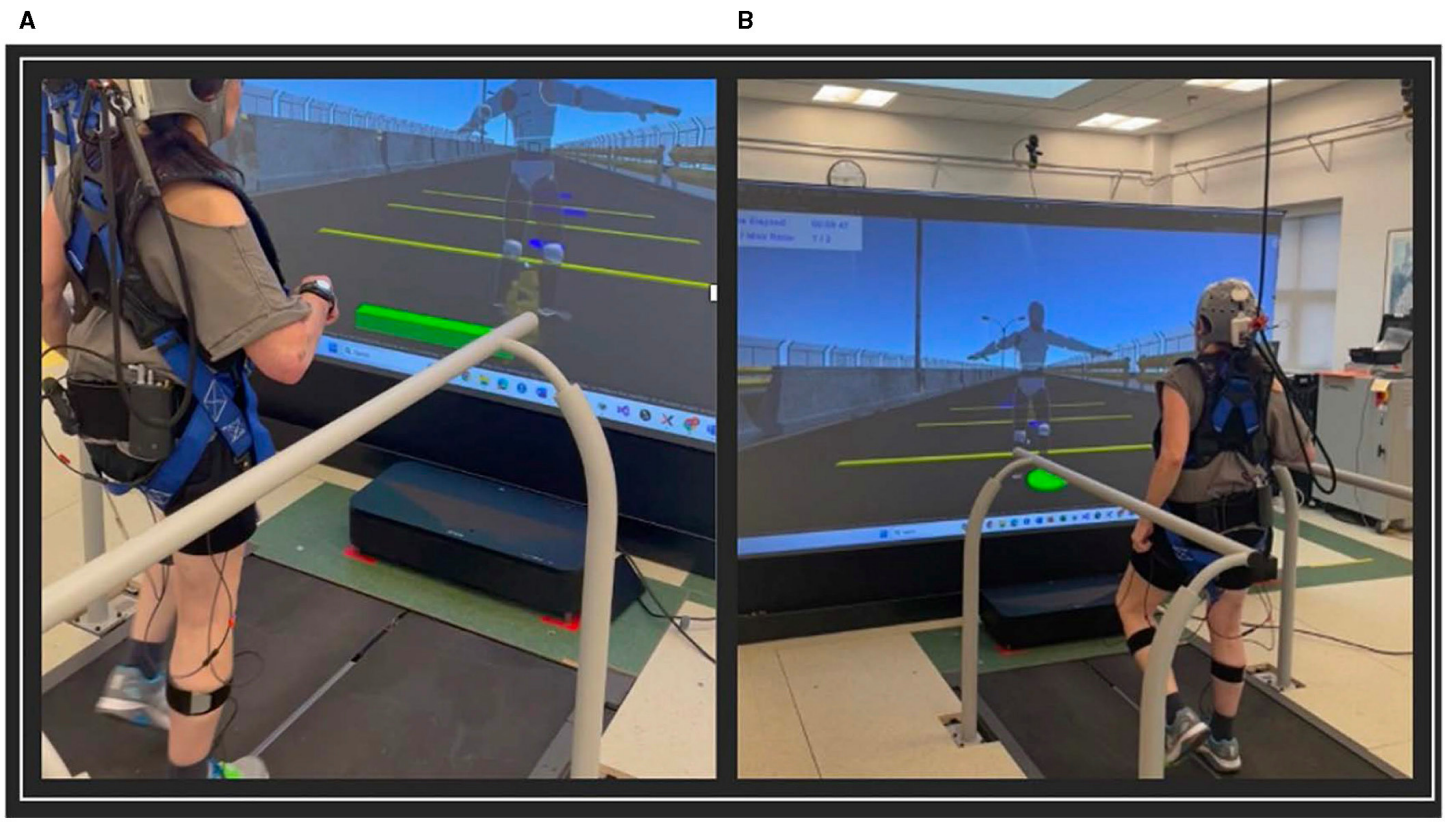
VR training game. **(A)** Stepping over rectangular obstacles; **(B)** Stepping on circular obstacles.

The VR game involved navigating a path with circular stepping-stones and rectangular obstacles presented at varying distances apart. The goal of the VR game was for the participant to step on the circular stones and step over the obstacles, while walking on the treadmill (Figure 1). Participants progressed through increasingly difficult levels as their training advanced. There was a total of 10 different levels and the difficulty of each level modified based on parameters such as obstacle speed, size, spacing, and frequency. Object location in the virtual world was scaled by the participant's proportional dimensions.

In each level, the number of obstacles that the participant navigated correctly (hits) and incorrectly (misses) was recorded. The participant's performance score was determined by the number of hits they completed over the number of obstacles they missed. The scores were displayed on the screen to motivate and encourage the participants A performance score of 70% was required to proceed to the next difficulty level on the next visit. Game levels were designed to provide a challenging but not frustrating environment.

### Training protocol

Participants walked on the in-ground split-belt treadmill (Bertec, Columbus, OH). VR training for each participant consisted of 30-min training sessions, 5 days a week for 2 consecutive weeks. Participants walked on the treadmill for three 10-min trials with at least a five5minute break in between trials. The virtual environment was generated in Unity 3D (Unity Technologies, San Francisco, CA) and displayed on a projector screen in front of the treadmill. Participants viewed an avatar guided by real-time performance feedback from Xsens inertial sensors (Movella, Henderson, NV) placed on the lower extremities, pelvis and sternum of the participant.

### Outcome measures

The evaluation of the training intervention on the improvement of gait function was measured through a series of clinical tests: Dynamic Gait Index (DGI) (Shumway-Cook and Woollacott, 2012) which assesses the ability to modify gait in response to task demands, the Berg Balance Scale (BBS) (Berg et al., 1992), which evaluates static and dynamic balance, indicating an individual's risk of falling and balance proficiency, the 10-meter Walk Test (10MWT) which measures preferred walking speed, an important indicator of physical health (Middleton et al., 2015) and the Timed Up and Go Test (TUG) (Podsiadlo and Richardson, 1991) to gage function mobility. Three trials of the TUG and 10MWT were done and the average value for the time and velocity were recorded for analysis. Changes in outcomes between baseline and post-intervention were calculated to determine if a functional change in gait and mobility followed the intervention. Changes in outcomes between the post-intervention assessment and the 2-week follow-up time point were calculated to assess if the improvements were maintained. Additionally, the mean change values were computed by group to evaluate the effect of the tDCS.

In assessing the efficacy of the intervention, it is crucial to ensure that any changes have meaningful clinical implications, therefore, Minimal Clinically Important Differences (MCIDs) were utilized for each outcome measure. MCIDs represent the smallest change in a score that patients perceive as beneficial, and which would suggest a change perceived as beneficial by patients and clinicians (Wells et al., 2001). For the DGI, a minimal detectable change ranges from 1.9 to 4 points for stroke patients (Romero et al., 2011; Lin et al., 2010; Jonsdottir and Cattaneo, 2007; Alghadir et al., 2018). For the TUG test, a 2.9–3.4 s between assessments scores is considered clinically meaningful (Gautschi et al., 2017; Wells et al., 2001; Flansbjer et al., 2005). A change in velocity of 0.14–0.16 m/s is considered the MCID for the 10MWT (Tilson et al., 2010; Perera et al., 2006) and the MCID for the BBS is 8 points (Berg et al., 1992; Wells et al., 2001).

## Case description

**Figure 2:**
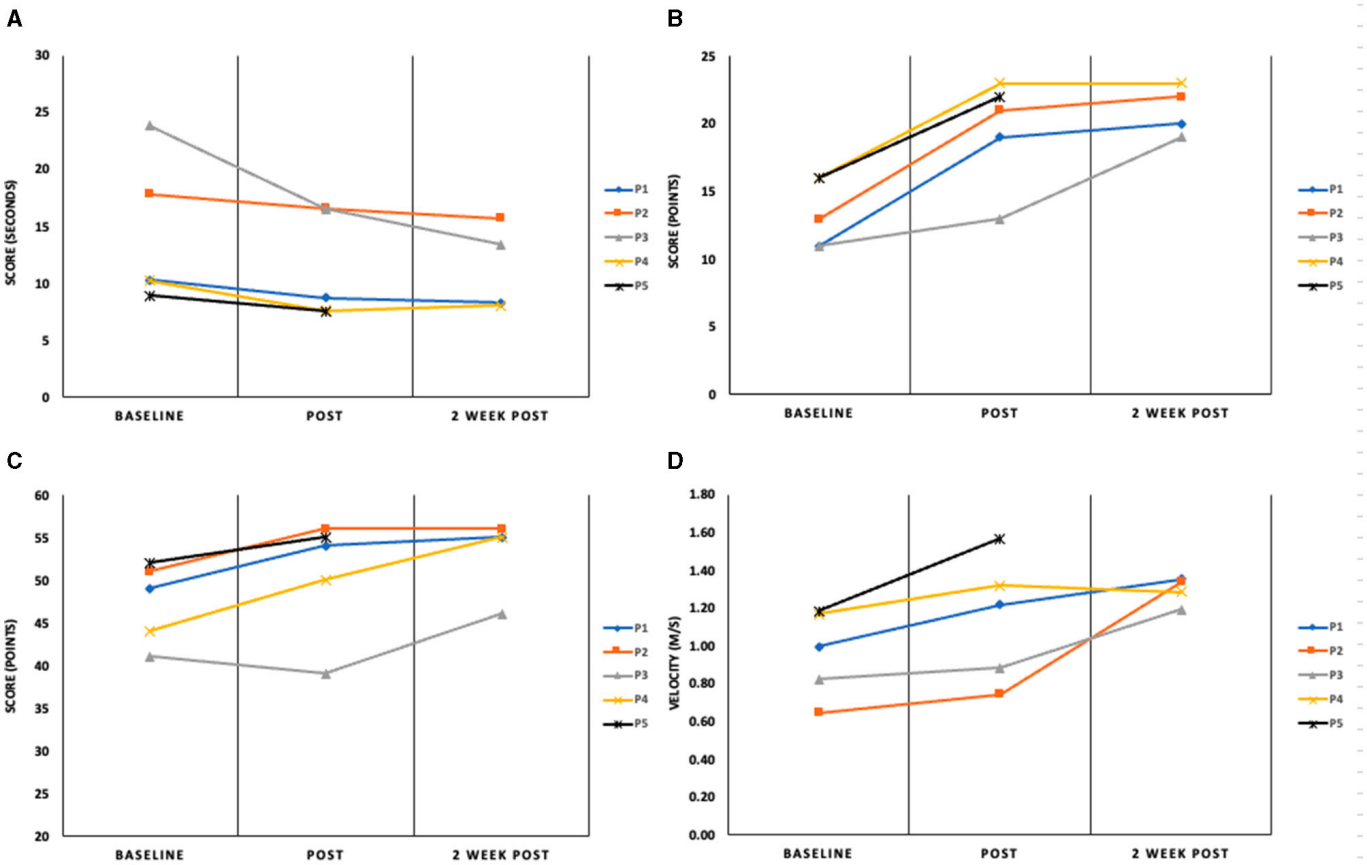
Assessment outcomes for the **(A)** timed Up and Go (seconds); **(B)** Dynamic Gait Index (points); **(C)** Berg Balance Scale (points) and **(D)** 10-m Walk Test (m/s) at baseline, immediately post-intervention and 2-week follow-up. P1–5 represent the four participants in this report.

The study included five participants who completed the invention protocol and clinical gait and mobility assessments. Improvements in performance are reported from baseline to post-intervention and at a 2-week follow up. Change in assessment scores from pre-intervention to immediate post and from immediate post to 2-week follow up are shown in Table 2, where clinical significance was found. Figure 2 illustrates the changes in the DGI and TUG. Performance scores and their correlation to the outcome measures can be found in the Supplementary Data.

**Table 2:** Changes in assessment scores by time points.

**a. ΔDGI Test Scores**	**ΔPost-Pre**		**Mean ΔPost- Pre by Group**	**Δ2wk − Post**
Sham	P1	8^*^		1^*^
	P2	8^*^	7.33	1^*^
	P5	6		+
tDCS	P3	2	4.5	6^*^
	P4	7		0
**b**. **ΔTUG Test Scores (s)**	**ΔPost-Pre**		**Mean** **ΔPost- Pre by Group**	**Δ2wk** **−** **Post**
Sham	P1	−1.57		−0.436
	P2	−1.28	−1.37	−0.836
	P5	−6.23^*^		+
tDCS	P3	−7.32^*^	−4.98	−3.12^*^
	P4	−2.62		0.45
**c**. **ΔBBS Test Scores**	**ΔPost-Pre**		**Mean** **ΔPost- Pre by Group**	**Δ2wk** **−** **Post**
Sham	P1	5	4.33	1
	P2	5		0
	P5	3		+
tDCS	P3	−2	2.00	7
	P4	6		5^*^
**d**. **Δ10MWT Test Scores (m/s)**	**ΔPost-Pre**		**Mean** **ΔPost- Pre by Group**	**Δ2wk** **−** **Post**
Sham	P1	0.22		0.14
	P2	0.10	0.234	0.59
	P5	0.38		+
tDCS	P3	0.06	0.10	0.31
	P4	0.15		−0.03

a. Dynamic Gait Index (points); b. Timed Up and Go (seconds); c. Berg Balance Scale (points); d. 10-meter Walk Test (m/s). P1–P5 represent the five participants.

^*^Clinically significant change from baseline; +withdrawn from study.

### Participant 1 (P1)

Participant 1, a 57-year-old female, is 10 years post hemorrhagic stroke in the left cerebral cortex with right hemiparesis and has diminished sensation on her affected side. After her stroke, P1 received physical, occupational and speech therapy. P1 is a community ambulator as evidenced by her baseline gait speed [1.00 m/s (Bowden et al., 2008)] and reports as a short community walker. Participant 1's mobility and functional gait scores were as follows: pre-intervention-10MWT: 1.00 m/s, BBS: 49, DGI: 11, TUG: 10.18s; post-intervention-10MWT: 1.22 m/s, BBS: 54, DGI: 19, TUG: 8.70s and at the two-week follow up-−10MWT: 1.35 m/s, BBS: 55, DGI: 20, TUG: 8.26s.

Improvements were seen in all measured outcomes from baseline to post-interventions. The DGI and 10MWT scores exceeded the clinically significant threshold suggesting increased confidence and potential for improved independence in activities of daily living (Lin et al., 2010; Romero et al., 2011; Shumway-Cook et al., 1997; Perera et al., 2006; Tilson et al., 2010). The gait speed increased above the clinical threshold at the 2-week follow-up (Δ0.36 m/s). Scores for all other measures at the two-week follow-up stayed consistent with the post-intervention values. Results for P1 should be considered because this individual was high-functioning and active. Although her stroke was 10 years prior, the intervention was able to affect changes in gait function.

### Participant 2 (P2)

Participant 2, a 61-year-old male, is 9 years post-ischemic stroke in the left cerebral cortex with right hemiparesis, wearing an AFO on his right leg. He continues speech therapy and has limited community ambulation (10MWT: 0.65 m/s at baseline). His mobility and gait scores were as follows: pre-intervention-10MWT: 0.65 m/s, BBS: 51, DGI: 13, TUG: 17.85s; post-intervention-10MWT: 0.75 m/s, BBS: 56, DGI: 21, TUG: 16.57s and at the 2-week follow up-10MWT: 1.34 m/s, BBS: 56, DGI: 22, TUG: 15.73s.

Like P1, P2 showed improvements in all clinical assessments immediately following the intervention. Clinically significant improvements from baseline were noted post-intervention for the DGI (+8 points) and the 2-week follow-up in 10MWT (+0.69 m/s). All other improvements held steady at the 2-week time point. The most notable result for P2 is the fact that significant changes in gait function as measured by the DGI were present after the intervention and were maintained for 2 weeks. This suggests a functional change in gait.

### Participant 3 (P3)

Participant 3 is a 44-year-old male who presents with right hemiparesis from a hemorrhagic stroke in the basal ganglia 20 years ago. He uses a cane to ambulate and wears an AFO on his right leg. P3 received physical, occupational and speech therapy after his stroke. Based on baseline gait speed (0.82 m/s), P3 is considered a limited community ambulator. His mobility and functional gait scores were as follows: pre-intervention-10MWT: 0.82 m/s, BBS: 41, DGI: 11, TUG: 23.83s; post-intervention-10MWT: 0.88 m/s, BBS: 39, DGI: 13, TUG: 16.51s and at the 2-week follow up-10MWT: 1.19 m/s, BBS: 46, DGI: 19, TUG: 13.39 s.

P3 had clinically significant increases in the 10MWT, the DGI test and TUG test. The gait speed measured for the 10MWT increased by 0.36 m/s at the 2-week follow-up assessment with virtually no increase at the post-intervention visit. A similar trend was found for the DGI—a small increase from baseline at the post-intervention assessment (+2 points) with clinical significance being achieved at the 2-week follow-up (+8 points from the baseline measurement). There was a clinically significant increase at both time points after the intervention for the TUG. The TUG scores greatly exceeded the clinically significant standards with an improvement of 7.32 s, from baseline to immediate post and 10.44 s from baseline to 2-week post-intervention (Gautschi et al., 2017; Podsiadlo and Richardson, 1991). P3 entered the study with a notably slow gait speed and limited control over fine motor movements in the lower extremities. This significant improvement suggests that even with substantial deficits, the VR intervention can lead to meaningful gains in functional mobility and gait speed. However, the marked deficits at the outset meant that any improvement, even if significant, may not translate into functional independence or a return to pre-stroke mobility levels.

### Participant 4 (P4)

Participant 4 is a 69-year-old female who suffered an ischemic stroke in the left hemisphere of her frontal lobe 12 years prior. She developed hemiparesis on her right side, affecting her motor abilities and functional independence. P4 is a community ambulator (baseline gait speed = 1.17 m/s). Participant 4's mobility and functional gait scores were as follows: pre-intervention-10MWT: 1.17 m/s, BBS: 44, DGI: 16, TUG: 10.23s; post-intervention-10MWT: 1.32 m/s, BBS: 50, DGI: 23, TUG: 7.61s and at the two-week follow up-10MWT: 1.28 m/s, BBS: 55, DGI: 23, TUG: 8.06s.

P4 showed improvements in all measured outcomes post-intervention. Most notably, the DGI score of 16 points at baseline was predictive of falls (Wrisley and Kumar, 2010). Post-intervention and the two-week follow-up scores for the DGI are indicative of safe ambulators (>19) (Shumway-Cook et al., 1997). The increase in DGI score of 7 points meets the threshold for clinical improvement (Lin et al., 2010). The BBS score increased by 6 points post-intervention and by another 5 points at the two-week follow-up assessment period. This represents a clinically significant change in static and dynamic balance. While the time recorded for the TUG test did decrease by 2.6 s and walking speed, as measured by the 10MWT did increase, neither of these scores met the criteria for a significant change. The results for P4 suggest successful improvements in gait function and mobility, likely contributing to increased independence during walking. Sustained improvements in all four measures demonstrated by P4, especially in the 2-week follow-up, indicate the lasting impact of the intervention beyond just short-term benefits.

### Participant 5 (P5)

Participant 5 is a 29-year-old male, seven years post-hemorrhagic stroke in the basal ganglia, with right hemiparesis and noticeable tremors in his right arm. He wears AFO on his right leg and walks with the aid of a service animal. P5 has undergone occupational and speech therapy and is currently in aquatic therapy once a week. P5 is community ambulator (baseline gait speed = 1.19 m/s). Participant 5's mobility and functional gait scores were as follows: pre-intervention-10MWT: 1.19 m/s, BBS: 52, DGI: 16, TUG: 8.87s; post-intervention-10MWT: 1.57 m/s, BBS: 55, DGI: 22, TUG: 7.51 s. P5 was withdrawn from the study at the 2-week follow-up due to an unrelated hospitalization and no data was recorded for the 2-week follow-up time point.

Improvement was noted in all clinical assessments immediately post-intervention; however, clinical significance was only achieved for the DGI [Δ Post-pre assessment-6 points (Lin et al., 2010)] and the 10MWT [Δ 0.38 m/s improvement (Perera et al., 2006)]. These results collectively indicate improvements in gait function and mobility. Of particular importance is the significant change in gait speed. The ability to ambulate at a speed that provides proper biomechanics is linked to health, function and quality of life (Middleton et al., 2015). Additionally, P5 found the immersive environment and the game-playing aspect of the intervention motivating and wanted to continue to improve his score.

## Discussion

This pilot case series demonstrates the potential of VR treadmill training in improving gait and mobility among chronic stroke survivors, with MCIDs found in key clinical assessments such as the 10MWT, DGI and TUG tests. All participants experienced improvements following the training sessions, regardless of whether they received tDCS or sham tDCS in combination with the VR therapy. Although differences were found between the experimental groups, it is difficult to conclude due to the small number of participants in each group (tDCS = 2; Sham = 3).

The extent of improvement in clinical assessments of dynamic balance and functional mobility varied among the participants. Results indicated MCIDs in DGI scores for three out of the four participants, suggesting enhanced gait functionality. Participant 3 achieved a MCID in TUG score suggesting a functional change in mobility. The variability in the data highlights the complexity of stroke rehabilitation and the need for individualized rehabilitation programs which can be delivered using VR technology.

Functional gains, even if they did not reach the threshold for the MCID, were maintained for 2 weeks after the completion of the intervention for all participants who were evaluated at 2 weeks post-intervention for the DGI, the BBS, the TUG and three out four participants for the 10MWT. To improve outcomes, further exploration into optimizing duration and intensity for sustained recovery after VR interventions is needed.

The study's small sample size across the two groups (sham vs. active tDCS) limits the ability to make broad conclusions about the effectiveness of the VR intervention. Additionally, participant variability—differences in time since stroke, stroke type and severity, lesion location, and pre-stroke functional abilities—influences baseline function and recovery potential. The study also did not control or account for external activities or additional rehabilitation efforts that the participants might have engaged with while participating, potentially confounding their results.

Overall, these findings emphasize the potential of the immersive and interactive nature of VR alone to be sufficient to drive meaningful clinical outcomes. Results suggest that VR may be a valuable tool for stroke rehabilitation, positively influencing participants' quality of life and daily functioning. Further studies should examine its combined use with anodal tDCS stimulation to assess whether this approach could improve treatment outcomes.

## Data Availability

The original contributions presented in the study are included in the article/Supplementary material, further inquiries can be directed to the corresponding author.
