# Supplementary material for: Anodal tDCS and virtual reality gait rehabilitation in individuals with chronic stroke: a case series report
Source: Front Stroke. 2025 Jan 30;4:1489031. doi: 10.3389/fstro.2025.1489031 (PMC12802614; doi:10.3389/fstro.2025.1489031)
Supplement: Supplementary file 1 [file Data_Sheet_1.pdf]

Supplementary Data

Table 1: Performance Scores

Participant	Visit #	Level	Walking Speed (m/s)	Performance Score (%)
1	1	x	x	x
	2	2	0.45	83.2
	3	3	0.45	76.5
	4	4	0.46	95.0
	5	5	0.50	86.5
	6	6	0.60	89.4
	7	7	0.62	90.9
	8	8	0.65	89.0
	9	9	0.65	98.6
	10	10	0.70	84.2
2	1	1	0.20	82.2
	2	2	0.28	88.0
	3	3	0.36	91.5
	4	4	0.40	85.8
	5	5	0.40	87.1
	6	6	0.42	90.7
	7	7	0.45	86.3
	8	8	0.50	61.0
	9	8	0.50	72.7
	10	9	0.53	88.3
3	1	1	0.66	22.7
	2	1	0.67	14.3
	3	1	0.67	6.0
	4	1	0.69	25.0
	5	1	0.69	15.0
	6	1	0.69	19.5
	7	1	0.70	7.3
	8	1	0.70	22.0
	9	1	0.70	17.0
	10	x	x	x
4	1	1	0.53	94.7
	2	2	0.56	92.7
	3	3	0.57	95.0
	4	4	0.58	95.3
	5	5	0.60	96.0
	6	6	0.62	94.3
	7	7	0.64	98.7
	8	8	0.66	96.0
	9	9	0.67	98.3
	10	10	0.68	90.7
5	1	1	0.75	74.4
	2	2	0.80	71.0
	3	3	0.85	55.9
	4	3	0.85	61.1
	5	3	0.85	61.4
	6	3	0.85	80.0
	7	4	0.85	70.4
	8	5	0.85	81.9
	9	6	0.85	88.8
	10	7	0.85	92.2

Supplementary Data

Table 1 shows the data collected during the VR training sessions for each level of the game completed by the participant. The initial speed at level 1 was based on the overground walking speed measured by the 10MWT and adjusted for each training level. The performance score is calculated by determining the percentage of obstacles correctly navigated divided by the total number of obstacles encountered for each session.

Table 2: Pearson Correlation with Mean Performance Score

Variable	r	p
**DGI**	0.905	0.035*
**ΔDGI**	0.954	0.120
**BBS**	0.854	0.066*
**ΔBBS**	0.992	<0.001**

DGI - Dynamic Gait Index; TUG – Timed Up & Go;
BBS – Berg Balance Scale; 10MWT – 10-meter Walk Test
*Significant at the 0.05 level
**Significant at the 0.01 level

Table 2 shows a Pearson correlation analysis conducted to examine the relationship between the mean performance score (calculated as the average score across all 10 levels) and the outcome measures evaluated at the post-assessment time point. The score and the change in the score from baseline were included in the analysis. Although the sample size is low, the DGI and the BBS variable met the assumptions of bivariate normality and linearity and were therefore included in the correlation analysis.
